# “Crack lung and heart” presenting after chemotherapy in a 65-year-old man with non-Hodgkin lymphoma

**DOI:** 10.3747/co.2008.202

**Published:** 2008-01

**Authors:** G.G. Alvarez, R.H.C. van der Jagt

**Affiliations:** * Department of Medicine, Division of Respirology, University of Ottawa, Ottawa, Ontario; † Department of Medicine, Division of Hematology, University of Ottawa, Ottawa, Ontario

**Keywords:** “Crack lung”, cocaine, non-Hodgkin lymphoma

## Abstract

Crack cocaine can cause a variety of pulmonary and cardiac complications. Pulmonary findings in a 65- year-old man with non-Hodgkin lymphoma who presented with shortness of breath not resolving with antibiotics are presented here. The usual manifestation of “crack lung” in an unusual clinical circumstance underlines the importance of a clinical history in such cases. The finding of “crack lung” preceded the diagnosis of probable “crack heart.” No other similar published case reports could be identified in the literature.

## 1. PRESENTATION

**FIGURE 1:**
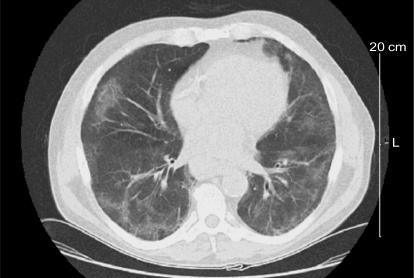
Computed tomography image of thorax, October 2005.

A 65-year-old man with biopsy-proven follicular non-Hodgkin lymphoma grade 2 was referred for treatment. At initial presentation, he had shortness of breath on exertion and wheezing. Past medical history included idiopathic venous thrombosis in his right arm, hypertension, and chronic sinusitis. He was a 20-packper-year smoker and a known cocaine abuser. He was taking amoxicillin, fluticasone propionate nasal spray, coumadin, and thiazide. He had no known drug allergies, no travel history, and no sick contacts. His chest radiograph demonstrated interstitial opacifications, and an axial computed tomography (ct) image of the thorax (Figure 1) showed bibasilar “ground glass” opacification with septal thickening.

This patient did not respond to a course of amoxicillin, and his shortness of breath began to worsen. Vital signs were normal. Physical exam demonstrated bilateral wheezing and extensive lymphadenopathy in the cervical, suboccipital, posterior cervical, anterior cervical, tonsillar, submental, supraclavicular, and femoral regions. The initial cardiac examination revealed normal heart sounds, with a normal jugular venous pressure, but with 2+ pitting edema bilaterally.

The patient was referred to respirology consultation for investigation to rule out an opportunistic infection. He missed the appointment with the respirologist and returned 4 months later to the clinic.

### 1.1 Investigations

The patient’s complete blood count at the first visit was completely normal. In addition, electrolytes, liver enzymes, urea, and creatinine were all within normal limits. Serology for hiv was negative. Sputum initially showed heavy growth of normal respiratory flora. No acid-fast bacilli were seen in the sputum. Bronchoscopy washings were negative for Gram and fungal stains. An enhanced ct of the thorax in the lung window confirmed bibasilar “ground glass” opacifications with septal thickening. Significant mediastinal lymphadenopathy was noted. A cardiac nuclear scan performed at the time revealed normal left ventricular function with a normal contractile pattern.

### 1.2 Course Between First and Second Thorax CT

The patient admitted to having a crack cocaine addiction. He had missed his first respirology appointment as a result of being referred to an addiction rehabilitation program. He received no treatment during the 4-month interval following the missed appointment. He had successfully remained off crack cocaine during the 4 months.

**FIGURE 2:**
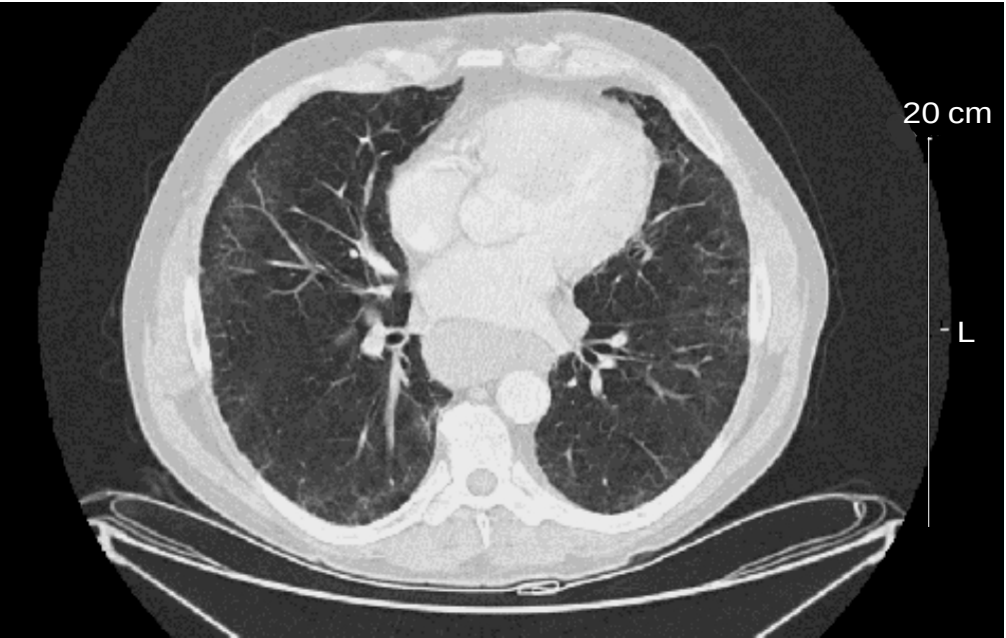
Computed tomography image of thorax, February 2006.

On repeat ct (Figure 2), the “ground glass” opacifications of 4 months past had almost completely disappeared (during this period, the patient was not smoking crack cocaine). Complete pulmonary function testing, including a CO diffusion test, was normal.

This patient unfortunately resumed his crack cocaine habit. He then developed anasarca. A subsequent two-dimensional echocardiogram revealed global systolic left ventricular dysfunction with an estimated ejection fraction of 25%–28%, and the patient was admitted to hospital for treatment of heart failure.

Previously, this man had been treated with 8 cycles of cyclophosphamide, rituximab, vincristine, and prednisone with only a partial response. The presence of active heart failure precluded most forms of applicable chemotherapy. He is now under re-evaluation for treatment options.

### 1.3 Diagnosis

A presumptive diagnosis of “crack lung” was made based on the distinctive pattern seen on thorax ct (multifocal “ground glass” attenuation associated with septal thickening)^1^. This condition improved without any intervention except cessation of crack cocaine use.

## 2. DISCUSSION

In Canada, increasing use of oral crack cocaine is a relatively neglected public health problem 1. Crack cocaine is the free base of cocaine hydrochloride. To obtain the “freebase” form, cocaine is boiled in water and dried 2. Precipitate is obtained by adding ether or alcohol 3. The precipitate is then heated to produce the final form of the drug. The name “crack cocaine” comes from the characteristic cracking sound that the precipitate makes when heated.

When crack cocaine is smoked, it enters the pulmonary circulation and reaches the cerebral circulation within 6 seconds (as compared with 18 seconds for intravenous cocaine and several minutes for inhaled powder cocaine) 3. This rapid onset of action, with its tachyphylaxis effect, eventually requires an increased dose to achieve the same effect, making the crack form of cocaine a highly addictive substance. Because crack cocaine is inhaled, most of its side effects are cardiopulmonary in nature.

Crack cocaine can produce a variety of pulmonary effects including acute respiratory symptoms, exacerbation of asthma, thermal airway injury, pneumothorax, and pneumomediastinum, cryptogenic organizing pneumonia, pulmonary hemorrhage, non-cardiogenic pulmonary edema, pulmonary infiltrates with eosinophilia or interstitial pneumonitis 3,4, and pulmonary infarction from embolus 5. Pulmonary function, including CO diffusion, does not seem to be deleteriously affected by the use of crack cocaine 6.

Our patient presented with dyspnea and the ct findings described earlier. The differential diagnosis included *Pneumocystis jiroveci*, cytomegalovirus, pulmonary hemorrhage, idiopathic interstitial pneumonia, hypersensitivity pneumonitis, and pulmonary edema.

Although smoking crack cocaine is supposed to increase susceptibility to pulmonary infection because of dysfunction of the pulmonary alveolar macrophages, a bronchoscopy did not reveal an infectious cause in our patient. Furthermore, bronchoscopy did not show any visual evidence of gross pulmonary hemorrhage. However, clinically unapparent alveolar hemorrhage can occur in healthy individuals who smoke crack cocaine 7.

The finding of significant left ventricular dysfunction on echocardiogram was felt to be most likely attributable to the crack cocaine. Cardiovascular events associated with crack cocaine include myocardial infarction, myocarditis, dilated cardiomyopathy, heart failure, coronary and peripheral vascular spasm, dysrhythmia, and sudden cardiac death 8.

Non-cardiogenic pulmonary edema was thought to be caused by increased alveolar permeability from exposure to crack cocaine; however, evidence suggests that, as compared with tobacco smokers and non-crack-and non-tobacco-smoking controls, no increase in alveolar permeability is evident 9.

Our patient received no chemotherapy agent that is generally thought to cause cardiac damage, although rituximab has been noted to cause immediate cardiotoxicity 10 and to occasionally cause delayed reduction in left ventricular function 11.

Our patient also had no burn marks on his hands, but in the presence of pulmonary infiltrates, evidence of burn marks on the hands caused by crack pipes used to smoke the cocaine should provide important clues to the diagnosis 4.

Crack cocaine abuse is uncharacteristic in the age group represented by our patient, but nonetheless should be kept in the differential when the “ground glass” pattern of opacities with septal thickening is seen on thorax ct.

## 3. CONCLUSIONS

“Ground glass” opacities on ct scan of the thorax in an immunocompromised host is a non-specific finding, with a wide differential; however, the present report highlights the fact that a careful history can sometimes provide valuable information to reach the diagnosis. This particular case also highlights the potential cardiac toxicity of repeated crack cocaine use. The increased use of this illegal drug in Canadian cities should alert clinicians to look for such manifestations in all age groups.

## References

[1] Gotway MB, Marder SR, Hanks DK (2002). Thoracic complications of illicit drug use: an organ system approach. Radiographics.

[2] Haydon E, Fischer B (2005). Crack use as a public health problem in Canada: call for and evaluation of “safer crack use kits”. Can J Public Health.

[3] Haim DY, Lippmann ML, Goldberg SK, Walkenstein MD (1995). The pulmonary complications of crack cocaine. A comprehensive review. Chest.

[4] Gatof D, Albert RK (2002). Bilateral thumb burns leading to the diagnosis of crack lung. Chest.

[5] Ramachandaran S, Khan AU, Dadaparvar S, Sherman MS (2004). Inhalation of crack cocaine can mimic pulmonary embolism. Clin Nucl Med.

[6] Kleerup EC, Koyal SN, Marques–Magallanes JA, Goldman MD, Tashkin DP (2002). Chronic and acute effects of “crack” cocaine on diffusing capacity, membrane diffusion, and pulmonary capillary blood volume in the lung. Chest.

[7] Baldwin GC, Choi R, Roth MD (2002). Evidence of chronic damage to the pulmonary microcirculation in habitual users of alkaloidal (“crack”) cocaine. Chest.

[8] Thadani PV (1996). nida conference report on cardiopulmonary complications of “crack” cocaine use. Clinical manifestations and pathophysiology. Chest.

[9] Tashkin DP, Kleerup EC, Hoh CK, Kim KJ, Webber MM, Gil E (1997). Effects of “crack” cocaine on pulmonary alveolar permeability. Chest.

[10] Garypidou V, Perifandis V, Theodoridou S (2004). Cardiac toxicity during rituximab administration. Leuk Lymphoma.

[11] Kanamori H, Tsutsumi Y, Mori A (2006). Delayed reduction in left ventricular function following treatment of non-Hodgkin’s lymphoma with chemotherapy and rituximab, unrelated to acute infusion reaction. Cardiology.

